# The *Carthamus tinctorius* L. and *Lepidium apetalum* Willd. Drug Pair Inhibits EndMT through the TGF*β*1/Snail Signaling Pathway in the Treatment of Myocardial Fibrosis

**DOI:** 10.1155/2023/6018375

**Published:** 2023-01-12

**Authors:** Zhou Zhou, Dufang Ma, Yu Zhou, Keke Zhang, Yang Liu, Zhen Wang, Yong Wang

**Affiliations:** ^1^First Clinical Medical College, Shandong University of Traditional Chinese Medicine, Jinan 250000, China; ^2^Affiliated Hospital of Shandong University of Traditional Chinese Medicine, Jinan 250000, China; ^3^First Clinical Medical College, Lanzhou University, Lanzhou 730000, China

## Abstract

**Background:**

Myocardial fibrosis (MF) is an essential pathological factor for heart failure. Previous studies have shown that the combination of *Carthamus tinctorius* L. and *Lepidium apetalum* Willd. (C-L), two types of Chinese herbal medicine, can ameliorate MF after myocardial infarction (MI) in rats and inhibit the activation of myocardial fibroblasts. However, the mechanism of C-L in the treatment of MF remains unclear.

**Methods:**

A rat model of MF with left anterior descending coronary ligation-induced MI was first established. Then, the effects of C-L on cardiac function, MF, and endothelial-to-mesenchymal transition (EndMT) were evaluated by the left ventricular ejection fraction (LVEF), serum N-terminal pro-brain natriuretic peptide (NT-proBNP) levels, Masson's trichrome staining, and immunohistochemical and immunofluorescence staining. Next, a hypoxia-induced cardiac microvascular endothelial cell (CMEC) model was established to observe the effects of C-L on EndMT. The supernatant of CMECs was collected and used to culture cardiac fibroblasts (CFs) and observe the effects of CMEC paracrine factors on CFs.

**Results:**

Animal experiments indicated that C-L improves the cardiac function of rats after MI, inhibits the progression of EndMT and MF, and downregulates TGF*β*1, Snail, and CTGF expression. Cell experiments showed that drug-loaded serum containing C-L inhibits the EndMT of CMECs under hypoxic conditions. The culture supernatant of CMECs grown under hypoxic conditions significantly activated CFs. After treatment with C-L, the activating factor for CFs in hypoxic CMEC culture supernatant was substantially downregulated, and the effect of the culture supernatant on CF activation was also reduced. However, TGF*β*1 agonists inhibited the effects of C-L on CMECs and CFs.

**Conclusion:**

Our data demonstrated that by regulating the TGF*β*1/Snail pathway, C-L inhibits EndMT of CMECs and reduces the release of CF-activating factors in cells undergoing EndMT.

## 1. Introduction

Heart failure (HF), a life-threatening condition, currently affects approximately 64 million people worldwide [1]. After a pathological myocardial injury, collagen fibers will replace the defective myocardium and exacerbate HF. Thus, inhibition or reversal of myocardial fibrosis (MF) has become an important strategy for treating HF [2, 3]. After pathological stimulation, fibroblasts transform into myofibroblasts, the major source of the extracellular matrix during MF, as these cells show significantly increased collagen synthesis [4]. Fibroblasts have been studied to ameliorate MF, but increasing evidence has shown that nonfibroblasts in the heart also play an important role in MF [5]. Under pathological conditions, nonfibroblasts, including cardiomyocytes and inflammatory cells, affect fibroblast activity and function by secreting factors that induce fibroblast activation [6, 7].

We previously reviewed the effects of cardiac microvascular endothelial cells (CMECs) on HF and concluded that CMECs also contribute to the progression of MF [8]. Under certain conditions (hypoxia, oxidative stress, inflammation, abnormal fluid shear stress, etc.), the phenotypic transformation of endothelial cells involves the loss of endothelial-specific markers, including platelet EC adhesion molecule-1 (CD31), Tie1, Tie2, VE-cadherin, and von Willebrand factor (VWF), as well as the acquisition of mesenchymal markers, including alpha smooth muscle actin (*α*-SMA), N-cadherin, and vimentin. This process is known as an endothelial-to-mesenchymal transition (EndMT) [9]. During this process, endothelial cells peel away and separate from the ordered endodermis, migrate into the parenchyma and lose their apical-basal polarity, becoming an essential part of the fibroblast pool during MF [10]. Studies have also shown that upregulation of the zinc finger protein SNAI1 (Snail) induced by transforming growth factor beta (TGF*β*) plays a crucial role in promoting EndMT in human umbilical vein endothelial cells (HUVECs) [11]. Upregulation of Snail not only promotes EndMT to generate a large number of fibroblasts but also induces fibroblasts to transition into myofibroblasts through connective tissue growth factor (CTGF) secreted by cells undergoing EndMT, which accelerates the development of MF [12]. However, the abovementioned studies were limited to HUVECs, and their applicability to CMECs is currently unknown. In addition, whether the TGF*β*1/Snail signaling pathway inhibits the activation of cardiac fibroblasts (CFs) by regulating the paracrine process of CMECs after EndMT has not been clarified.

Herbs and their main components are widely used in disease treatment worldwide [13]. Recently, many studies have confirmed the potential value of herbal medicine [14–16]. Traditional Chinese medicine (TCM) has been used to treat HF for thousands of years. In TCM theory, HF belongs to the “cardiac edema” category, which was first noted in the Eastern Han dynasty in Zhang Zhongjing's Synopsis of Golden Chamber (Jin Gui Yao Lue). Dr. Zhang noted that the pathological basis of cardiac edema is “blood vessel stasis and obstruction caused by water pathogens attacking the heart.” As a result, the TCM treatment of HF should “activate blood” and “promote urination.” *Carthamus tinctorius* L. (Asteraceae, Chinese Pinyin: Hong Hua), which functions as a blood activator, was first reported in the Synopsis of Golden Chamber and is often used by TCM doctors to promote blood circulation and expel blood stasis. *Lepidium apetalum* Willd. (Cruciferae, Chinese Pinyin: Ting Li Zi) is believed to promote diuresis and resolve edema, which was recorded in the Compendium of Materia Medica (Ben Cao Gang Mu) of the Ming dynasty. Both *C. tinctorius* L. and *L. apetalum* Willd., are used to treat HF, especially in combination [7, 17]. In TCM theory, using two herbs in combination for the same therapeutic purpose is referred to as a “drug pair.” However, the pharmacodynamic properties and critical targets of the *C. tinctorius* L. and *L. apetalum* Willd. drug pair (C-L) remain unclear.

Here, we studied the regulatory effect of C-L on EndMT of CMECs for the first time and revealed the effect of profibrotic factors secreted by cells undergoing EndMT on CF activation.

## 2. Materials and Methods

### 2.1. Chemicals and Reagents

A Masson's trichrome staining kit (G1006), hematoxylin and eosin (H&E) staining kit (Cat# G1005), Cell Counting Kit-8 (CCK-8) kit (Cat# G4103), 4′,6-diamidino-2-phenylindole (DAPI) dihydrochloride staining kit, FITC-conjugated goat anti-rabbit IgG (H + L) (Cat# GB22303), and Cy3-conjugated goat anti-rabbit IgG (H + L) (Cat# GB21303) were purchased from Wuhan Servicebio Biotechnology Co., Ltd. (Wuhan, China). Rabbit anti-rat glyceraldehyde-3-phosphate dehydrogenase (GAPDH) (Cat# ab181602), anti-Snail (Cat# ab180714), anti-TGF*β*1 (Cat# ab179695), anti-CTGF (Cat# ab6992) and anti-Smad2/3 (Cat# ab202445) antibodies were purchased from Abcam, Inc., (Cambridge, MA, USA). Rabbit anti-rat CD31 (Cat# GB11063-2), anti-vimentin (Cat# GB111308), anti-VWF (Cat# GB11020), and anti-*α*-SMA (Cat# GB111364) antibodies were purchased from Wuhan Servicebio Biotechnology Co., Ltd. A secondary antibody for western blotting and immunohistochemical staining, goat anti-rabbit IgG H&L (Cat# ab205718), was purchased from Abcam, Inc. (Cambridge, MA, USA). The SPARKeasy Improved Tissue/Cell RNA Kit (Cat# AC0202), SPARKscript II RT Plus Kit (Cat# AG0304), and 2 × SYBR Green qPCR Mix (Cat# AH0103-B) kit were purchased from Shandong Sparkjade Biotechnology Co., Ltd. (Shandong, China). N-terminal pro-brain natriuretic peptide (NT-proBNP) (Cat# JYM0213Ra) enzyme-linked immunosorbent assay (ELISA) kits were purchased from Wuhan ColourfulGene Biological Technology Co., Ltd. (Wuhan, China). CTGF (Cat# E-EL-R0259c) and TGF*β*1 (Cat# E-EL-0162c) ELISA kits were purchased from Elabscience Biotechnology Co., Ltd. (Wuhan, China). An Enhanced Bicinchoninic Acid (BCA) Protein Assay Kit (Cat# P0010S) was purchased from Beyotime Biotechnology Co., Ltd. (Shanghai, China). Radioimmunoprecipitation assay (RIPA) lysis buffer was purchased from Dalian Meilun Biotechnology Co., Ltd. (Dalian, China). SRI-011381 (Cat# HY-100347A, purity: 99.58%, an activator of TGF*β*1) was purchased from MedChemExpress Co., Ltd. (New Jersey, USA).

### 2.2. Ultrahigh Performance Liquid Chromatography (UHPLC) Coupled with a Quadrupole-Orbitrap Mass Spectrometer

A total of 100 *μ*L of 0.5 g/mL C-L solution (see “2.8 preparation of the herbal solution” for the preparation method) was diluted with water ten times [high-performance liquid chromatography (HPLC) grade]. Next, 200 *μ*L of diluent and 10 *μ*L of internal standard (L-2-chlorophenylalanine, 0.06 mg/mL; prepared by methanol) were added and filtered with a 0.22-*µ*m membrane. Then, the sample was centrifuged at 8,000 × *g* for 5 min, and the supernatant was subsequently extracted for analysis. The component spectrum was analyzed using the Dionex Ultimate 3000 RS UHPLC) system together with a Q-Exactive plus quadrupole-Orbitrap mass spectrometer equipped with a heated electrospray ionization (ESI) source (Thermo Fisher Scientific, Waltham, MA, USA) in the ESI-positive and ESI-negative ion patterns. The Acquity UPLC HSS T3 column also includes positive and negative modes. Finally, the separation was completed based on a binary gradient elution system including water and acetonitrile according to the following protocol: 0 min, 5% acetonitrile; 2 min, 5% acetonitrile; 4 min, 25% acetonitrile; 8 min, 50% acetonitrile; 10 min, 80% acetonitrile; 14 min, 100% acetonitrile; 15 min, 100% acetonitrile; 15.1 min, 5% acetonitrile; and 16 min, 5% acetonitrile. The column's flow rate and temperature were set to 0.35 mL/min and 45°C, respectively. During the analysis, all operations were performed at 4°C. C-L components were identified based on the mass spectrometric data of the standard substance by reviewing the references.

### 2.3. Preparation of C-L Herbal Solution

The C-L herbal solution included a 3 : 5 extracted mixture of particles of *C. tinctorius* L. (Jiangyin Tianjiang Pharmaceutical Co., Ltd., No. 21011561) and *L. apetalum* Willd., (Jiangyin Tianjiang Pharmaceutical Co., Ltd., No. 20120911). The drugs were dissolved in 0.9% saline solution (NS) and stored at 4°C.

### 2.4. Animal and Postinfarction HF Models

Sprague–Dawley (SD) male rats (specific-pathogen free) weighing 190–210 g were purchased from Beijing Charles River Laboratories (Beijing, China, Certificate No. 2021-0006). The care and use of laboratory animals in this study followed the Guidelines for the Care and Use of Laboratory Animals (National Institutes of Health publication, 8^th^ edition, 2011). All operation procedures were reviewed and approved by the Animal Ethics Committee of the Affiliated Hospital of Shandong University of Traditional Chinese Medicine (Permit number: 2021-33). All rats were raised in a room with constant temperature, humidity control, and a 12-h light/dark cycle. Standard food and water were also provided.

The rats were randomly divided into 3 groups: a control group (*n* = 10), a sham operation group (*n* = 10), and a myocardial infarction (MI) model group. The rats in the model group underwent left anterior descending branch (LAD) ligation, as documented in the references, to construct the MI model [18]. Specifically, SD rats were anesthetized and ventilated by a ventilator (Harvard Apparatus). After the chest was opened and the heart was exposed, the LAD was ligated with a 7.0 surgical suture proximal to its main branching point. Changes in the electrocardiogram's ST segment were used to assess the presence of MI. The rats in the sham operation group (sham) underwent a similar procedure without actual ligation in the LAD. One week after surgery, the rats' cardiac function was assessed with a Vetus 7 (Mindray, China) system equipped with a P10-4s and 14–18 MHz imaging transducer. Rats meeting the criteria of cardiac insufficiency [left ventricular ejection fraction (LVEF) < 50%] were included in the study (*n* = 40) and randomly divided into 4 groups: the HF model, low-dose, medium-dose, and high-dose groups. Then, the rats in the high-, medium-and low-dose C-L groups were treated with low, medium, and high C-L doses, respectively, by intragastric administration. Based on the human and animal surface area of the equivalent dose conversion ratio table, the C-L doses in the low, medium, and high doses of C-L groups were 4.8, 2.4, and 1.2 g/kg/day, respectively [19]. The rats in the other groups received an equal volume of 0.9% sodium chloride solution. After four weeks of treatment, the rats were anesthetized with 2% pentobarbital sodium to obtain blood from the abdominal aorta in each group, and the left ventricular myocardium was also collected for the study.

### 2.5. Serum NT-proBNP

Blood samples were collected from the abdominal aorta and allowed to stand for 2 h. The serum was collected after centrifugation. A biotin double antibody sandwich ELISA was used to determine the concentration of NT-proBNP in the serum. The absorbance was measured at 450 nm using a Multiskan GO1510 microplate reader (Thermo Fisher Scientific, USA).

### 2.6. Echocardiographic Evaluation

Echocardiographic measurements were performed at the end of the study period. Left ventricular function was assessed by LVEF and ventricular wall motion. All measurements were performed by an investigator blinded to the experimental groups.

### 2.7. Masson's Trichrome Staining and Immunohistochemical Analysis

The left ventricular tissues were dehydrated and embedded in paraffin, sliced into 4 *μ*m sections, and mounted on glass slides. Then, the slices underwent dewaxing and hydration, hematoxylin staining, Ponceau S staining, phosphomolybdic acid hydrate staining, aniline blue staining, dehydration, and sectioning. The sections were observed under a light microscope.

### 2.8. Preparation of Rat Drug-Loaded Serum Containing C-L

Twenty male SD rats were used to prepare drug-loaded serum containing C-L. In short, the rats were intragastrically administered a moderate dose of C-L solution (2.4 g/kg/day). On the 7^th^ day, blood was collected from the abdominal aorta in a sterile environment 2 h after administration. Then, the serum was separated and inactivated in a 55°C water bath for 15 min and stored at −20°C for future use.

### 2.9. Cell Culture

CMECs (CP-R135) and CFs (CP-R074) were purchased from Procell Life Science & Technology Co., Ltd. (Wuhan, China). CMECs and CFs were cultured in CM-R135 (Procell, Wuhan, China) and CM-R074 (Procell, Wuhan, China), respectively, at 37°C in a humidified atmosphere with 5% CO_2_. The control group CMECs were cultured under these conditions until various indices were assessed.

For the establishment of an MI model in vitro, a hypoxic cell model was used in our study. Before different treatments were performed, the CMECs, which were grown to approximately 80% confluency, were placed in a hypoxia incubation chamber with 95% N_2_ and 5% CO_2_. The incubation time was 48 h, and all experiments were conducted in triplicate. After a hypoxic intervention, the hypoxic cells were randomly divided into 4 groups: the hypoxia group and the low-dose, medium-dose, and high-dose C-L groups. The drug-loaded serum containing C-L (C-L DS) was added to the culture medium of the low-, medium-, and high-dose groups and diluted to low (2.5%), medium (5%), and high (10%) concentrations, respectively. Subsequently, to study the molecular mechanism by which C-L improves MF, we cultured CMECs again and divided them into 4 groups: the control, hypoxia, C-L treatment, and SRI-011381 groups. The cells in the control group were cultured under conventional conditions, and the cells of the other groups were used to construct a hypoxia model for 48 h. Then, the CMECs in the C-L treatment group were treated with 10% C-L DS. The cells in the SRI-011381 group was additionally treated with 10 *μ*M SRI-011381 [dissolved in dimethyl sulfoxide (DMSO) and then further attenuated with cell culture medium to the consistency required for in vitro experiments]. The treatment duration was 24 h. After treatment, the culture supernatant from the CMECs in each group was collected and used to culture CFs for 24 h.

### 2.10. CF Viability Assay

CF viability was examined with CCK-8 assays according to the manufacturer's protocols. Briefly, 100 *µ*L of the cell suspension was seeded in a 96-well plate (5,000 cells per well), and then, the experimental treatment was performed after the cells reached confluence. The culture medium, which contained CCK-8 reagent and 10% base solution, was placed in an incubator for 2 h, and the absorbance was measured at 450 nm.

### 2.11. Immunofluorescence and Immunohistochemical Staining

Immunofluorescence staining for VWF, CD31, and *α*-SMA was performed in tissues and cells. Monolayer CMECs were grown on cover glasses and used in successive experiments. After treatment, the cells were fixed with 4% paraformaldehyde for 10 min at 4°C and incubated with 2% bovine serum albumin for 1 h. The cells were incubated with anti-*α*-SMA or anti-CD31 antibodies followed by secondary antibodies. Rat left ventricles were fixed in 4% paraformaldehyde, embedded in paraffin blocks, and cut into 4-*μ*m slices. Dewaxed slices were blocked with 1% bovine serum albumin and incubated with anti-VWF and anti-*α*-SMA antibodies followed by secondary antibodies. The nuclei were stained with DAPI. Primary antibodies against VWF, CD31, and *α*-SMA were diluted to 1 : 400, 1 : 500, and 1 : 500, respectively. The goat antirabbit IgG secondary antibodies were diluted to 1 : 10000. Images were captured by a Ni-V fluorescence microscope (Nikon, Japan). The fluorescence intensity was analyzed by ImageJ (version 1.8.0). CD31 immunohistochemical staining of tissues was performed. Dewaxed slices were blocked with 2% bovine serum albumin, incubated with an anti-CD31 primary antibody, and then incubated with a secondary antibody. Mayer's hematoxylin was used to counterstain the slices. Images were captured by a NanoZoomer S60 Digital Slide Scanner. The positive area was analyzed by ImageJ (version 1.8.0).

### 2.12. ELISA Analysis

TGF*β*1 and CTGF in the culture supernatant of CMECs were determined by ELISA kits according to the manufacturer's instructions and analyzed with a Multiskan GO1510 microplate reader (Thermo Fisher Scientific, USA).

### 2.13. mRNA Analysis

mRNA levels were quantified by real-time polymerase chain reaction (PCR) with the LightCycler® 480II platform (Roche, Switzerland) and the ^ΔΔ^CT method. Total RNA was collected with the SPARKeasy Improved Tissue/Cell RNA Kit according to the manufacturer's instructions. cDNA was synthesized by the SPARKscript II RT Plus Kit. Quantitative PCR was performed by the LightCycler® 480II Real-Time PCR Detection System. All primers were synthesized by Integrated DNA Technology (Servicebio, China). The primer sequences for mRNA analysis were as follows: TGF*β*1 forward 5′-AGGAGACGGAATACAGGGCT-3′ and reverse 5′-CCACGTAGTAGACGATGGGC-3′; Snail forward 5′-GCAGAGTGCCTTTGTACCCT-3′ and reverse 5′-CCACTTGGCCCCTAACAAGT-3′; CTGF forward 5′-ACTGTTGGCGAACAAATGGC-3′ and reverse 5′-CTGCCTCCCAAACCAGTCAT-3′; COL1A1 forward 5′-GGAGAGAGCATGACCGATGG-3′ and reverse 5′-GGTGGGAGGGAACCAGATTG-3′; COL3A1 forward 5′-TTCCTGGGAGAAATGGCGAC-3′ and reverse 5′-ACCAGCTGGGCCTTTGATAC-3′; GAPDH forward 5′-GCATCTTCTTGTGCAGTGCC-3′ and reverse 5′-GATGGTGATGGGTTTCCCGT-3′; actin forward 5′-CTCTGTGTGGATTGGGCT-3′ and reverse 5′-CGCAGCTCAGTAACAGTCCG-3′. GAPDH or actin expression was used to normalize gene expression.

### 2.14. Western Blotting Analysis

Myocardial tissues (50 mg) or cells (5 × 10^5^) from each group were homogenized and lysed in RIPA lysis buffer, and the protein concentration was determined by a BCA kit. Normalized proteins were loaded on 10% sodium dodecyl sulfate-polyacrylamide gel electrophoresis (SDS-PAGE) gels and transferred onto a polyvinylidene fluoride (PVDF) membrane. Next, 5% skim milk was used to block the membrane, and the membrane was incubated with primary antibodies at 4°C overnight. Then, the membrane was incubated with a secondary antibody. Western blot analysis was conducted by a FluorChem FC3 Gel Imager System (ProteinSimple, USA). The signal density was quantified by ImageJ (version 1.8.0).

### 2.15. Statistical Analysis and Diagram Generation

All data were analyzed by GraphPad Prism 7 and are expressed as the mean ± standard deviation (SD). The significance of differences among three or more experimental groups was calculated by one-way analysis of variance. A value of *p* < 0.05 was considered statistically significant. Diagrams were generated using GraphPad Prism 7 and https://www.bioinformatics.com.cn/, a free online data analysis and visualization platform.

## 3. Results

### 3.1. Total Ion Flow Diagram of C-L by LC-MS/MS

**Figure 1:**
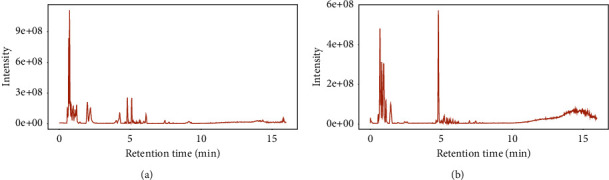
Total ion chromatograms (TICs) from C-L. (a) TIC (+ESI TCC) of C-L. (b) TIC (−ESI TCC) of C-L.

Qualitative analysis of the C-L components was performed using liquid chromatography-tandem mass spectrometry (LC-MS/MS). Chromatograms of total ions in ESI positive-ion and negative-ion modes for C-L are shown in Figures 1(a) and 1(b). We preliminarily identified 30 active components in the C-L solution under the screening condition of a score >50. The active components included cassiaside B; safflomin A; ciceritol; L-proline; neocarthamin; 3,6-diglucopyranosyl-4′,5,7-trihydroxyflavone; graveobioside B; pyroglutamic acid; galactaric acid; maesopsin 6-glucoside; allolactose; phloretin 2′-O-glucuronide; sudachiin A; perilloside B; 6-hydroxykaempferol 3,6-diglucoside 7-glucuronide; aromadendrin 3,7-diglucoside; 3-hydroxy-4-butanolide; quercetin 3-glucosyl-(1->2)-galactosyl-(1->2)-glucoside; L-arginine; genistin; pipecolic acid; linamarin; moracetin; quercetin 3-(2G-rhamnosylgentiobioside); tetrahydropentoxyline; isogenistein 7-glucoside; L-2-amino-3-(1-pyrazolyl) propanoic acid; geranylcitronellol; quercetin 3-O-glucosyl-rutinoside; and galactopinitol B. The specific information for the main components is shown in Table 1 of the Supplementary Materials.

### 3.2. C-L Improves Rat Cardiac Function after MI

**Figure 2:**
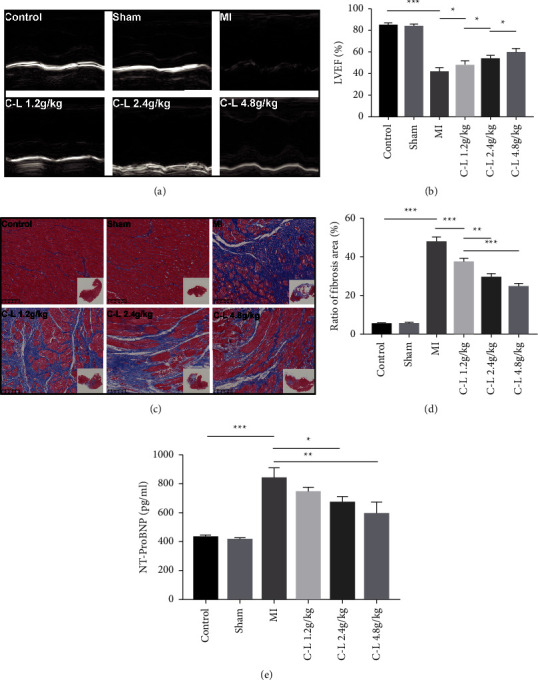
Evaluation of cardiac function and MF. (a, b) Echocardiography of the rats in each group after MI. (c, d) The effects of C-L on MF in the rats after MI were evaluated by Masson staining (×200). (e) Effects of C-L on serum NT-proBNP levels of the rats in each group. Data are presented as the means ± SDs. ^*∗*^*p* < 0.05, ^*∗∗*^*p* < 0.01, ^*∗∗∗*^*p* < 0.001.

After 28 days of C-L treatment, cardiac function and serum NT-proBNP levels of the rats with MI were assessed, and the degree of left ventricular fibrosis was evaluated in the pathological sections. The results indicated that the LVEF of the rats in the MI group was significantly reduced compared to that of the rats in the control group, and echocardiography revealed that the anterior wall of the heart (left ventricular wall) of the rats in the model group was significantly weakened (Figures 2(a)and 2(b)). The serum NT-proBNP level of the model group rats significantly increased (Figure 2(e)). However, no significant difference was observed between the sham and control groups. For Masson staining, the myocardium of the rats in the control and sham operation groups was bright red without a prominent fibrotic area.

In contrast, the myocardium of the rats in the model group had a large area of fibrotic lesions (blue area). These findings indicated that a large amount of collagen fiber synthesis occurred in the rat myocardium after MI (Figures 2(c) and 2(d)). However, the rats treated with C-L showed a significant improvement in LVEF, ventricular wall motion, NT-proBNP expression, and degree of MF. The effect was most significant in the high-dose group (Figure 2(e)). Although the level of NT-proBNP in the low-dose group rats was not significantly different from that in the model group, the LVEF and MF area substantially improved.

### 3.3. C-L Protects the Myocardium and CMECs in the Rats with MI

**Figure 3:**
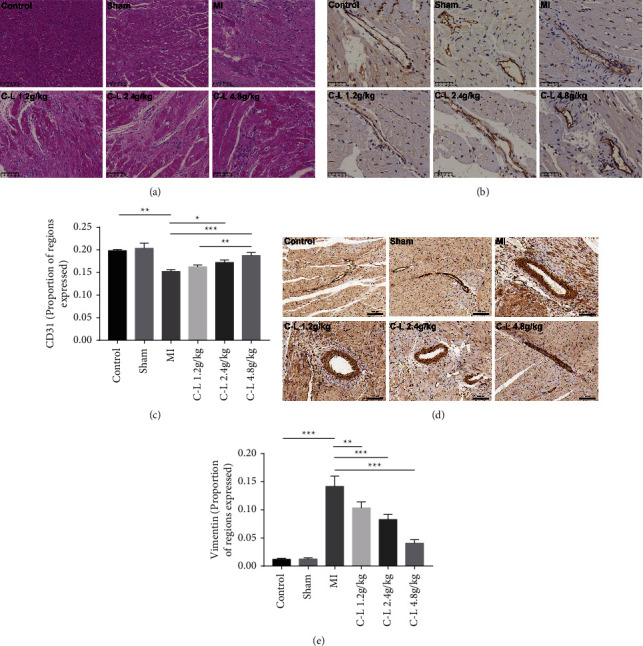
H&E and immunohistochemical staining. (a) H&E staining of rat myocardium (×200). (b, c) Immunohistochemical staining and quantitative evaluation of CD31 in rat myocardium (×400, quantified by ImageJ software). (d, e) Immunohistochemical staining and quantitative evaluation of vimentin in rat myocardium (×200, scale bar = 100 *μ*m, quantified by ImageJ software). ^*∗*^*p* < 0.05; ^*∗∗*^*p* < 0.01; ^*∗∗∗*^*p* < 0.001.

To determine the effect of C-L treatment on myocardial tissue, we used H&E staining to assess myocardial tissue damage. The H&E staining results indicated that the myocardial tissue of the control and sham operation groups was orderly and that the cell morphology was normal. In contrast, the myocardial tissue of the rats with MI showed a large amount of scar tissue, and myocardial cells exhibited disordered arrangements. After C-L treatment, the scar tissue in the rat myocardium was significantly reduced, and the regularity and cell integrity of the myocardium was substantially improved (Figure 3(a)). CD31 is a specific marker of endothelial cells, and vimentin is a marker of fibroblasts. In immunohistochemical staining, significant expression of CD31 on microvessels in the control and sham groups was noted, but vimentin staining was negative (Figures 3(b)–3(e)). However, CD31 expression was significantly decreased after MI, whereas vimentin expression was strongly positive. These results suggested that the EndMT process exists in myocardial microvascular endothelial cells after MI, but this process was significantly inhibited after C-L treatment.

### 3.4. C-L Inhibits EndMT in CMECs

**Figure 4:**
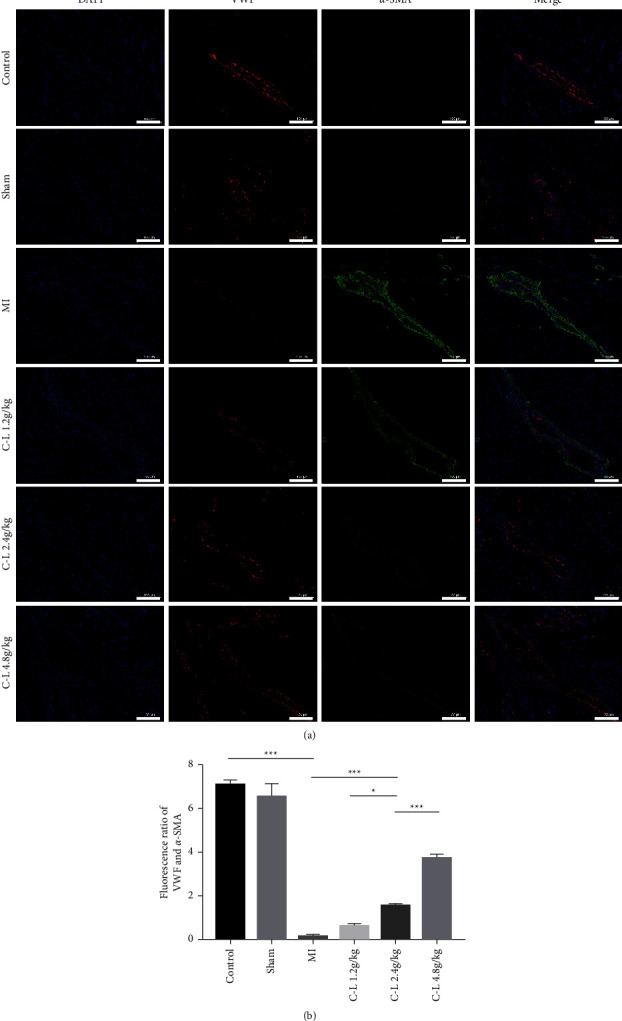
Immunofluorescence staining of rat myocardium. (a) Immunofluorescence staining of VWF and *α*-SMA in rat myocardium (×100, scale bar = 100 *μ*m). (b) Fluorescence ratio of VWF and *α*-SMA (quantitative analysis by ImageJ). ^*∗*^*p* < 0.05, ^*∗∗*^*p* < 0.01, ^*∗∗∗*^*p* < 0.001.

**Figure 5:**
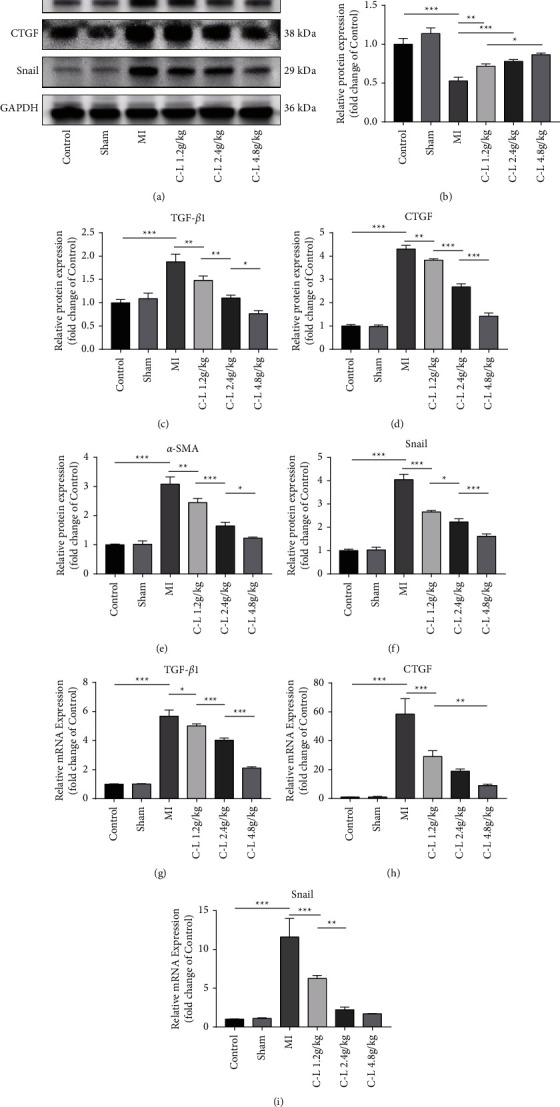
Western blot and qRT-PCR analysis of rat myocardium. (a–f) Images and quantitative results of western blots (quantitative analysis by ImageJ). (g–i) Results of qRT-PCR (the ^ΔΔ^CT method was used to calculate relative expression levels). ^*∗*^*p* < 0.05; ^*∗∗*^*p* < 0.01; ^*∗∗∗*^*p* < 0.001.

Recent studies have shown that EndMT-derived CFs play an essential role in the progression of MF [20]. VWF and *α*-SMA are specific markers of endothelial cells and mesenchymal cells (mainly fibroblasts in the myocardium). Immunofluorescence staining was used to evaluate the EndMT process of CMECs. The results indicated that VWF fluorescence (red) was strongly positive and that *α*-SMA fluorescence (green) was negative in the myocardial microvessels of the control group and the sham operation group (Figure 4(a)). However, the fluorescence of myocardial microvessels in the rats with MI showed the opposite pattern to that in the control group, and the fluorescence ratio of VWF and *α*-SMA was significantly lower than that of the control group (Figure 4). After the C-L treatment, the fluorescence ratio of VWF and *α*-SMA dramatically increased in the low-dose, medium-dose, and high-dose groups. These results suggested that the myocardium of the rats with MI undergoes significant EndMT. This process results in the pathological conversion of CMECs to CFs. Moreover, C-L significantly inhibited the EndMT of CMECs. These results are consistent with the detection of CD31 and *α*-SMA protein expression in rat myocardial tissue (Figure 5(a)).

### 3.5. C-L Inhibits EndMT and the Expression of Profibrotic Factors

TGF*β*1-mediated upregulation of Snail has been identified as a critical factor in EndMT and promotes the progression of tissue fibrosis. In addition, Snail overexpression promotes the secretion of CTGF in cells undergoing EndMT and the transformation of fibroblasts into myofibroblasts. We assessed the expression of TGF*β*1, Snail, CTGF, CD31, and *α*-SMA in the rat myocardium by western blotting. The results indicated that TGF*β*1, Snail, CTGF, and *α*-SMA expression was significantly upregulated in the rats with MI, whereas CD31 expression was inhibited (Figures 5(a)–5(f)). In addition, quantitative reverse transcription-polymerase chain reaction (qRT-PCR) indicated that TGF*β*1, Snail, and CTGF were highly expressed at the transcriptional level (Figures 5(g)–5(i)). The obtained results revealed high expression of profibrotic factors and a large area of endothelial cell injury in the myocardium of the rats after MI. However, in the myocardium of the rats treated with C-L, all the above-mentioned effects were inhibited, and the inhibitory effect correlated with the dose of C-L. Therefore, we hypothesized that the inhibitory effect of C-L on MF and EndMT is related to the regulation of TGF*β*1, Snail, and CTGF expression.

### 3.6. C-L Inhibits EndMT in CMECs

**Figure 6:**
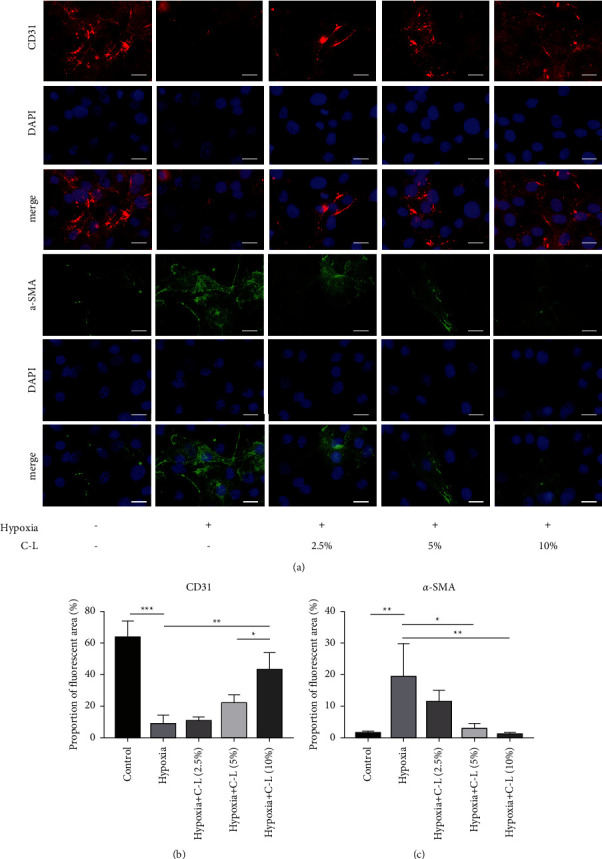
Immunofluorescence staining of CMECs. (a) CD31 and *α*-SMA immunofluorescence staining of CMECs (×400, scale bar = 50 *μ*m). (b, c) Area of CD31 and *α*-SMA fluorescence (quantitative analysis by ImageJ). C-L, drug-loaded serum containing C-L. ^*∗*^*p* < 0.05; ^*∗∗*^*p* < 0.01; ^*∗∗∗*^*p* < 0.001.

**Figure 7:**
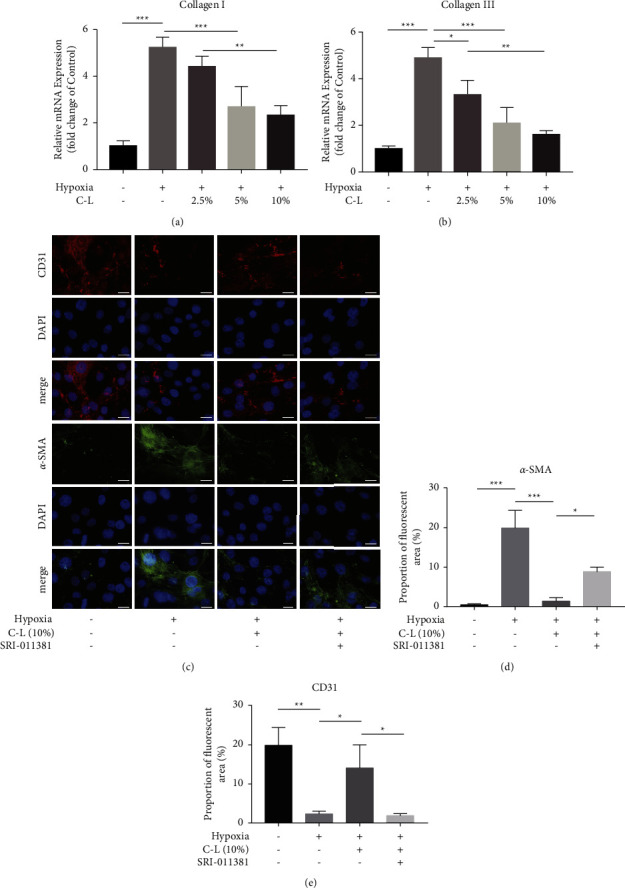
qRT-PCR and immunofluorescence staining results. (a, b) mRNA expression levels of type I and III collagen. (c) CD31 and *α*-SMA immunofluorescence staining of CMECs (×400, scale bar = 50 *μ*m). (d, e) Area of CD31 and *α*-SMA fluorescence (quantitative analysis by ImageJ). C-L, drug-loaded serum containing C-L. ^*∗*^*p* < 0.05; ^*∗∗*^*p* < 0.01; ^*∗∗∗*^*p* < 0.001.

Hypoxia after MI is an essential inducer of MF [21]. We constructed a hypoxic model of CMECs to simulate the environment of myocardial tissue after MI and observed the effect of C-L on EndMT by CD31 and *α*-SMA immunofluorescence staining of CMECs. As shown in Figures 6(a)–6(c), the CMECs of the control group in the normal culture exhibited strongly positive CD31 fluorescence staining and negative *α*-SMA fluorescence staining, which is a feature of endothelial cells. However, CD31 fluorescence in CMECs was weakened under hypoxic conditions, whereas positive *α*-SMA fluorescence was observed. These results indicated the existence of EndMT under hypoxic conditions. Notably, this hypoxia-induced change in CD31 and *α*-SMA expression was inhibited in the CMECs treated with drug-loaded serum containing C-L. In addition, we assessed the mRNA expression of type I collagen and type III collagen in CMECs by qRT-PCR. The mRNA expression levels of type I collagen and type III collagen in CMECs significantly increased under hypoxic conditions but were significantly reduced in the CMECs treated with C-L compared with those in the model group (Figures 7(a) and 7(b)). These results suggested that hypoxia activates the EndMT of CMECs, which can transform CMECs into mesenchymal cells (mainly fibroblasts) and enhance collagen synthesis. However, C-L inhibits the EndMT process and collagen synthesis of CMECs.

### 3.7. C-L Inhibits EndMT in CMECs via the TGF*β*1/Snail Pathway

To confirm that C-L ameliorates EndMT by inhibiting the TGF*β*1/Snail pathway, we added SRI-011381 (an activator of TGF*β*1) [22] to the culture medium of hypoxic CMECs treated with the optimal therapeutic concentration (10%) of drug-loaded serum containing C-L. Immunofluorescence staining of CD31 and *α*-SMA in CMECs showed that enhancement of the TGF*β*1/Snail pathway significantly inhibited the anti-EndMT effect of C-L on CMECs under hypoxic conditions (Figures 7(c)–7(e)). These results indicated that the C-L-mediated inhibition of EndMT in CMECs under hypoxic conditions is dependent on the inhibition of the TGF*β*1/Snail signaling pathway.

**Figure 8:**
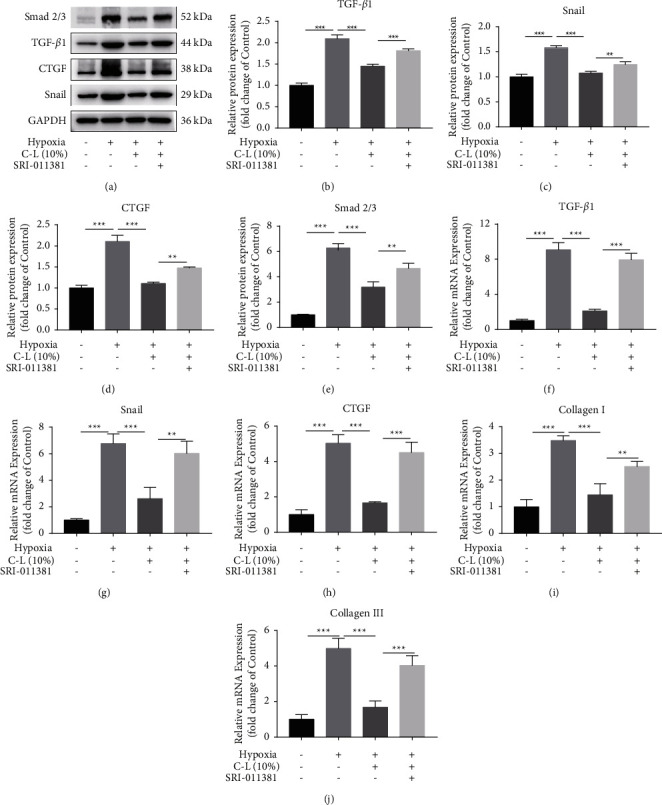
Western blot and qRT-PCR results of CMECs. (a–h) Protein and mRNA analysis of Smad1/2, TGF*β*1, Snail, and CTGF in CMECs. (i, j) The mRNA expression of type I and type III collagen in CMECs. C-L, drug-loaded serum containing C-L. ^*∗*^*p* < 0.05; ^*∗∗*^*p* < 0.01; ^*∗∗∗*^*p* < 0.001.

Previous studies have shown that the Smad signaling pathway is essential for EndMT [23]. Therefore, we also examined Smad2/3 expression. Smad2/3, TGF*β*1, Snail, and CTGF protein expression levels were assessed by western blotting. TGF*β*1, Snail, and CTGF mRNA expression levels were assessed by qRT-PCR. The results indicated that C-L strongly inhibits the upregulated expression of Smad2/3, TGF*β*1, Snail, and CTGF induced by hypoxia in CMECs. However, after the addition of a TGF*β*1 activator, this effect of C-L was attenuated (Figures 8(a)–8(h)). Moreover, the high collagen mRNA expression in CMECs under hypoxic conditions was inhibited by C-L, and this effect was weakened after the upregulation of TGF*β*1 (Figures 8(i) and 8(j)).

### 3.8. C-L Can Inhibit the Effect of CMECs on CF Activation

**Figure 9:**
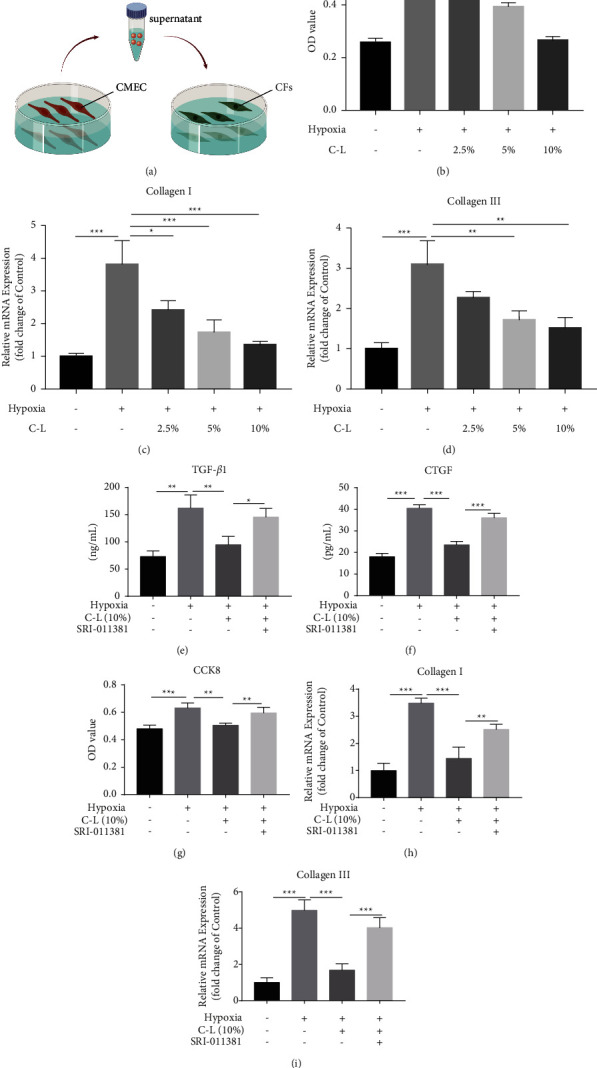
Effect of C-L on the culture supernatant of CMECs. (a) Schematic diagram of CF culture. (b) The proliferative activity of CFs was detected by the CCK-8 method. (c, d) Effect of the culture supernatant of different CMECs on the collagen synthesis of CFs. (e, f) The contents of TGF*β*1 and CTGF in the culture supernatant of CMECs were determined by ELISAs. (g) The proliferative activity of fibroblasts was detected by the CCK-8 method. (h, i) Type I and type III collagen mRNA expression in fibroblasts. C-L, drug-loaded serum containing C-L. ^*∗*^*p* < 0.05; ^*∗∗*^*p* < 0.01; ^*∗∗∗*^*p* < 0.001.

During CF promotion of MF, collagen fiber synthesis in the activated cells (myofibroblasts) was multiplied several times compared with that under normal conditions; consequently, myofibroblasts are the main effector cells of MF [24]. We collected the culture supernatant of each group of CMECs from the previous experiment, added them to the culture medium of CFs, and observed the effect on the proliferation and collagen synthesis of CFs (Figure 9(a)). The proliferative activity of CFs was assessed using CCK-8 assays, and we found that the culture supernatant of CMECs under hypoxic conditions could significantly increase CF proliferation (Figure 9(b)). In addition, qRT-PCR indicated that the culture supernatant of the hypoxia-treated CMECs could substantially increase the transcription level of the CF collagen gene (Figures 9(c) and 9(d)). However, the culture supernatant of the CMECs in the control group did not exhibit these effects, indicating that CMECs under hypoxia could secrete some profibrotic factors into the culture medium, driving the activation of CFs. Nevertheless, this promotion of CF activation was significantly inhibited in the culture supernatant of the CMECs treated with C-L. Therefore, we hypothesized that C-L inhibits the activation of CFs by inhibiting the paracrine pathway of CMECs.

### 3.9. C-L Inhibition of Paracrine-Mediated CF Activation by the TGF*β*1/Snail Pathway

We measured TGF*β*1 and CTGF levels in the cell supernatant using ELISAs to evaluate the ability of CMECs to secrete profibrotic factors. The results indicated that the levels of TGF*β*1 and CTGF significantly increased in the supernatant of CMECs under hypoxic conditions and that C-L significantly inhibited the secretion of profibrotic factors by CMECs. This inhibitory effect was strongly weakened after activating the TGF*β*1/Snail pathway (Figures 9(e) and 9(f)). Therefore, we believe that C-L inhibits the secretion of profibrotic factors by CMECs under hypoxic conditions by inhibiting the TGF*β*1/Snail pathway.

We also observed that the ability of CMECs to secrete profibrotic factors was regulated by TGF*β*1/Snail and that C-L inhibited the activation of CFs mediated by the culture supernatant of hypoxia-treated CMECs. Therefore, the culture supernatant of CMECs in the SRI-011381 group and other groups was collected to treat CFs, and we investigated whether TGF*β*1/Snail activation affected this inhibitory effect. We found that the culture supernatant of the hypoxia-treated CMECs stimulated the activation of CFs and that C-L inhibited this activation. However, the effect of C-L was significantly weakened after the enhancement of the TGF*β*1/Snail pathway (Figures 9(g)–9(i)). These results indicated that C-L inhibits the paracrine effect of fibroblast activation factors in CMECs by inhibiting the TGF*β*1/Snail pathway under hypoxic conditions and ultimately inhibits CF activation.

## 4. Discussion

MF is a compensatory process induced by high levels of myocardial cell death after MI. This process primarily fills the areas of the ventricular wall destroyed by partial myocardial death [25, 26]. However, the pathological scar tissue formed by MF exhibits poor relaxation and contraction abilities as well as poor compliance. Many fibrotic lesions promote the remodeling of the ventricular wall, enlarge the heart cavity, and seriously affect the systolic function of the ventricular wall, ultimately aggravating the progression of HF. Therefore, antifibrotic therapy is critical for improving cardiac insufficiency. Currently, some anti-MF drugs have been applied in clinical practice. However, regardless of whether the drugs have been used clinically for a long time (spironolactone, lisinopril, etc.) or are undergoing assessment in preliminary clinical trials (pirfenidone, doxycycline, etc.), their efficacy in reducing the formation of myocardial fibers is limited [27, 28]. Therefore, we need to further explore the pathological mechanism of MF and identify new and effective anti-MF strategies to achieve the clinical goal of effective control, even the reversal of HF.

*C. tinctorius* L. and *L. apetalum* Willd. are derived from the Luhong formula, a commonly used prescription in the treatment of HF, based on the TCM concept of drug pairs. Our previous clinical results indicated that the combination of the Luhong formula with the standardized treatment of modern medicine significantly improved the Minnesota Living with Heart Failure Questionnaire (MLHFQ) score of patients and significantly inhibited the serum levels of profibrotic factors (TGF*β*1, CTGF) and collagen (type I collagen and type III collagen) [29, 30]. By assessing profibrogenic factors in infarcted myocardial tissue, we found that TGF*β*1, CTGF, and TIMP1 expression in the myocardium of the rats with MI treated with the Luhong formula was substantially downregulated, and all three of these factors could promote fibroblast activation [31–34].

To further explore the anti-MF mechanism of the Luhong formula, we selected the C-L drug pair for pathological study. The results indicated that C-L significantly inhibited the degree of MF both in mice after MI and in rats after angiotensin II (Ang II) treatment, and the mechanism was related to the inhibition of TGF*β*1 expression. Notably, Ang II increased the proliferation of endothelial cells and CFs and promoted TGF*β*1 secretion in both types of cells, but this effect was inhibited using C-L [35, 36]. Previous studies have shown that Ang II enhances the proliferative activity of endothelial cells by promoting EndMT. In addition, CTGF secretion activates CFs, but this effect mainly depends on the autocrine release of CTGF by fibroblasts rather than other nonfibroblasts [33, 37]. In this study, we demonstrated that C-L could significantly inhibit EndMT in CMECs from the rats with MI, reduce the extracellular secretion of profibrotic factors (TGF*β*1 and CTGF) from EndMT-derived cells, and reduce the cellular activity and collagen synthesis of EndMT-derived cells and normal CFs. These results suggested that the mechanism by which C-L inhibits MF is closely related to EndMT and the paracrine pathways that regulate CMECs.

In addition to their role in protecting blood vessels, CMECs are closely related to MF. After stimulation with hypoxia or inducible factors, activated CMECs express adhesion factors that recruit inflammatory cells in circulating blood and secrete cytokines, as well as promote the infiltration of inflammatory cells and MF [38]. In addition, the transformation of microvascular endothelial cells into fibroblasts and the main mesenchymal cells of the myocardium are important pathological mechanisms of MF [39]. The transcriptional repressor Snail is a member of the zinc finger protein family and mainly inhibits the transcription of target genes by binding to specific sites in promoters of target genes after entering the nucleus [40]. Under certain conditions, endothelial cells transition into mesenchymal cells (EndMT) regulated by Snail [11]. Snail activation induces endothelial cells to transition into mesenchymal cells (especially fibroblasts), and EndMT-derived CFs synthesize a large amount of collagen, ultimately resulting in pathological collagen fiber deposition and fibrotic lesions. This process has also been confirmed in other studies on MF [12, 41]. TGF*β*1, a cytokine that can strongly promote fibrosis, was shown to play a critical role in various fibrotic diseases, including MF [42]. Recent studies have shown that Snail expression is regulated by TGF*β*1. Myocardin-related transcription factor (MRTF-B) is upregulated by TGF*β*1, and it binds to an SP1 element (SP1near) in the promoter of Snail to promote Snail expression, and Snail continues to induce EndMT [20, 43]. Thus, by inhibiting the TGF*β*1/Snail pathway, EndMT inhibition is an important therapeutic strategy for MF.

**Figure 10:**
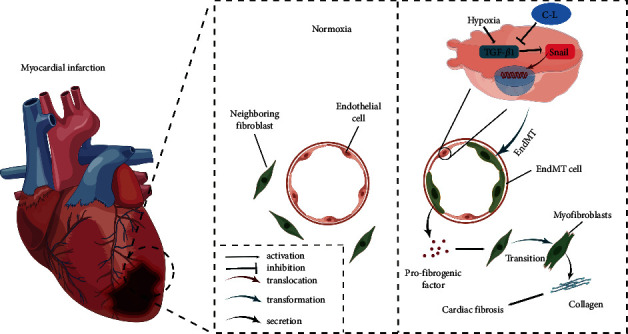
Schematic diagram of C-L treatment of MF. C-L effectively blocked the TGF*β*1/Snail pathway, inhibited EndMT and profibrogenic factor release in CMECs, and eventually attenuated hypoxia-induced MF.

However, current studies have not investigated whether the TGF*β*1/Snail pathway promotes the activation of CFs through paracrine signaling in addition to promoting EndMT. In this study, we found that C-L can significantly inhibit the degree of EndMT, decrease the collagen synthesis of cells undergoing EndMT and CFs, and reduce the activation of CFs. These effects were related to TGF*β*1 and Snail expression levels in the CMECs and were significantly inhibited during C-L treatment. However, after the addition of a TGF*β*1 activator in hypoxic CMECs, the antagonistic effect of C-L against EndMT was strongly inhibited via upregulation of TGF*β*1 and Snail, and the inhibition of collagen synthesis was also weakened. In addition, we found that the ability of C-L to inhibit CF activation was weakened by the enhancement of the TGF*β*1/Snail pathway. Therefore, we believe that C-L inhibits EndMT of CMECs and the paracrine pathway of cells undergoing EndMT that promotes the activation of CFs, playing a role in the treatment of MF, and this phenomenon is achieved by regulating the TGF*β*1/Snail signaling pathway (Figure 10).

## 5. Conclusion

In summary, we found that TGF*β*1/Snail pathway-mediated EndMT is a core mechanism of C-L therapy for MF. Experimental studies showed that C-L regulates the EndMT process by inhibiting the TGF*β*1/Snail signaling pathway and inhibits the activation of CFs induced by EndMT by regulating the paracrine pathway, ultimately playing an antifibrotic role. This study further elucidated the pharmacological mechanism of C-L in treating MF in HF and provided a new strategy for the treatment of MF. Nevertheless, our study has limitations. CMECs promote the activation of CFs through various factors in the paracrine pathway; however, we did not determine whether CTGF is a crucial factor. In addition, the results were not further verified by in vivo gene knockout experiments, and the main pharmacological components of C-L have not been determined. In future work, to discover the main components of C-L that ameliorate MF, we will provide more rigorous results using gene knockout animal models in accordance with pharmacological studies on various components contained in C-L.

## Data Availability

The original contributions presented in the study are included in the article/supplementary material. Further inquiries can be directed to the corresponding author.

## Supplementary Materials

Supplementary Materials Table 1: main components of C-L in UHPLC analysis.
