# Supplementary material for: The Carthamus tinctorius L. and Lepidium apetalum Willd. Drug Pair Inhibits EndMT through the TGFβ1/Snail Signaling Pathway in the Treatment of Myocardial Fibrosis
Source: Evid Based Complement Alternat Med. 2023 Jan 12;2023:6018375. doi: 10.1155/2023/6018375 (PMC9851799; doi:10.1155/2023/6018375)
Supplement: Supplementary Materials — Table 1: main components of C-L in UHPLC analysis. [file 6018375.f1.docx]

**Table 1** Main components of C-L in UHPLC analysis

| Nub | m/z | Retention time (min) | Ion mode | Formula | Name | Mean intensity | Score |
| --- | --- | --- | --- | --- | --- | --- | --- |
| 1 | 611.1619 | 4.79825 | neg | C26H30O14 | Cassiaside B | 40707428 | 56.1 |
| 2 | 613.1752 | 4.7916 | pos | C27H32O16 | Safflomin A | 17032732 | 55.1 |
| 3 | 563.183 | 0.76085 | neg | C19H34O16 | Ciceritol | 12156784 | 52.7 |
| 4 | 116.0707 | 0.727433 | pos | C5H9NO2 | L-Proline | 3709173 | 51.2 |
| 5 | 451.1226 | 4.806483 | pos | C21H22O11 | Neocarthamin | 2686713 | 55.6 |
| 6 | 595.1649 | 5.190417 | pos | C27H30O15 | 3,6-Diglucopyranosyl-4',5,7-trihydroxyflavone | 2468699 | 54.4 |
| 7 | 593.1518 | 5.169467 | neg | C27H30O15 | Graveobioside B | 2160462 | 51.6 |
| 8 | 147.0761 | 0.671833 | pos | C5H7NO3 | Pyroglutamic acid | 1631773 | 50.7 |
| 9 | 209.0295 | 0.70485 | neg | C6H10O8 | Galactaric acid | 1613455 | 50.8 |
| 10 | 451.1226 | 2.47915 | pos | C21H22O11 | Maesopsin 6-glucoside | 1510207 | 52.4 |
| 11 | 387.1145 | 0.732783 | neg | C12H22O11 | Allolactose | 1459041 | 53.6 |
| 12 | 451.1229 | 5.219283 | pos | C21H22O11 | Phloretin 2'-O-glucuronide | 1159379 | 51.3 |
| 13 | 1043.269 | 5.372683 | neg | C24H26O13 | Sudachiin A | 1027672 | 52.3 |
| 14 | 373.1504 | 5.571317 | neg | C16H24O7 | Perilloside B | 965790 | 53.6 |
| 15 | 801.1741 | 4.6103 | neg | C33H38O23 | 6-Hydroxykaempferol 3,6-diglucoside 7-glucuronide | 908670.1 | 51.8 |
| 16 | 613.1751 | 2.47915 | pos | C27H32O16 | Aromadendrin 3,7-diglucoside | 903601.1 | 53.2 |
| 17 | 282.1177 | 0.699617 | pos | C10H16O8 | 3-Hydroxy-4-butanolide | 854694.1 | 53.8 |
| 18 | 789.2067 | 4.641067 | pos | C33H40O22 | Quercetin 3-glucosyl-(1->2)-galactosyl-(1->2)-glucoside | 751126.7 | 57.6 |
| 19 | 175.1187 | 0.616267 | pos | C6H14N4O2 | L-Arginine | 699889.7 | 51 |
| 20 | 433.1121 | 2.60565 | pos | C21H20O10 | Genistin | 606420.2 | 51.8 |
| 21 | 130.0862 | 0.810933 | pos | C6H11NO2 | Pipecolic acid | 570770 | 51.7 |
| 22 | 248.1123 | 0.685733 | pos | C10H17NO6 | Linamarin | 570470.4 | 51.8 |
| 23 | 787.1946 | 4.627617 | neg | C33H40O22 | Moracetin | 567708.5 | 56.4 |
| 24 | 773.2119 | 5.126717 | pos | C33H40O21 | Quercetin 3-(2G-rhamnosylgentiobioside) | 543587.2 | 53.2 |
| 25 | 367.1493 | 3.756267 | pos | C17H22N2O7 | Tetrahydropentoxyline | 542328.3 | 50.5 |
| 26 | 433.1123 | 5.190417 | pos | C21H20O10 | Isogenistein 7-glucoside | 525837.2 | 51.8 |
| 27 | 156.0765 | 0.616267 | pos | C6H9N3O2 | L-2-Amino-3-(1-pyrazolyl)propanoic acid | 480786 | 53.8 |
| 28 | 310.3096 | 14.66443 | pos | C20H36O | Geranylcitronellol | 477686.6 | 51.5 |
| 29 | 771.1996 | 5.123033 | neg | C33H40O21 | Quercetin 3-O-glucosyl-rutinoside | 463621.1 | 54.1 |
| 30 | 339.1278 | 0.810933 | pos | C13H24O11 | Galactopinitol B | 455180.3 | 50.7 |
